# Association between Thyroid Function and Nonalcoholic Fatty Liver Disease in Euthyroid Type 2 Diabetes Patients

**DOI:** 10.1155/2020/6538208

**Published:** 2020-09-05

**Authors:** Bin Huang, Shengju Yang, Shandong Ye

**Affiliations:** Department of Endocrinology, The First Affiliated Hospital of USTC, Division of Life Science and Medicine, University of Science and Technology of China, Hefei, Anhui 230001, China

## Abstract

Thyroid function and type 2 diabetes mellitus (T2DM) are both associated with increased risks of adverse clinical outcomes in nonalcoholic fatty liver disease (NAFLD). Our study is aimed at evaluating the association between thyroid function and NAFLD in T2DM patients with normal thyroid function (euthyroid) and analyzing the potential effects of metformin on the pathological process. Overall, 369 T2DM patients were enrolled between July 2017 and September 2018 and stratified into NAFLD and non-NAFLD groups. Data on age, gender, body mass index (BMI, kg/m^2^), metformin use, and basal metabolic rate (BMR) were obtained from participants' records. All patients were tested for biochemical markers, indexes of glucose metabolism, lipid metabolism, bone metabolism, and thyroid function at baseline. Multivariate analyses detected increased odds of NAFLD among individuals with T2DM per unit increase in their BMI and free triiodothyronine (FT3) and thyroid stimulating hormone (TSH); the odds ratios (OR) were 1.25, 3.02, and 1.58, respectively (all *p* < 0.05). Positive correlations were detected between alanine aminotransferase (ALT) and FT3 (*r* = 0.221, *p* = 0.010), and negative correlations were noted between TSH and BMR (*r* = −0.618, *p* < 0.001) and between BMR and FT3 (*r* = −0.452, *p* < 0.001) in T2DM subjects with NAFLD. A significant difference in serum FT3 (*t* = 2.468, *p* = 0.0167) and TSH (*t* = 2.658, *p* = 0.010) levels was found between obese individuals with NAFLD who used and did not use metformin. The pathological mechanism of T2DM complicated by NAFLD in euthyroid patients may be associated with insulin resistance and a thyroid hormone resistance-like manifestation, i.e., relevant hypothyroidism. Metformin can potentially decrease the double-resistance situation, especially in obese individuals.

## 1. Introduction

Type 2 diabetes mellitus (T2DM), one of the largest epidemics the world has confronted, is often implicated in multiple organ dysfunctions, including liver, kidney, and cardiovascular diseases [1]. The primary effect of T2DM is insulin resistance, which causes accumulation of liver triglycerides due to a decreased response to insulin and subsequently impaired suppression of lipolysis [2]. The incidence of nonalcoholic fatty liver disease (NAFLD) in patients with T2DM is very high, and these patients have an increased risk of adverse clinical outcomes and death [3]. Existing clinical practice guidelines for NAFLD management do not provide specific recommendations for the therapy of T2DM patients with NAFLD.

It is well known that thyroid hormones have a significant effect on hepatic lipid metabolism [4]. Hypothyroidism-induced NAFLD has generally been attributed to interruptions in thyroid hormone (TH) signals, leading to reduced utilization of lipids by the liver [5]. Indeed, subclinical hypothyroidism, even in the upper range of normal serum thyroid stimulating hormone (TSH) concentrations, has been found to be associated with NAFLD in a dose-dependent way [6]. Kim et al. found that subclinical hypothyroidism and low-normal thyroid function are independent predictors of nonalcoholic steatohepatitis (NASH) and advanced fibrosis [7]. A study involving 20,289 euthyroid individuals with suspected NAFLD showed higher levels of the thyroid hormones free triiodothyronine (FT3) and TSH compared with individuals without NAFLD and confirmed the existence of a thyroid hormone resistance-like manifestation of NAFLD [8]. So far, however, there has been little research on the association between thyroid metabolism and NAFLD in a T2DM environment, which involves a more severe level of insulin resistance.

Metformin, the most commonly used group of insulin-sensitizing agents in T2DM treatments, has been demonstrated to have a potential TSH-lowering effect in euthyroid patients [9]. Since both thyroid function and T2DM are associated with increased risk of harmful clinical outcomes among individuals with NAFLD, we conducted this study with two aims: (1) to evaluate the association between thyroid function and NAFLD in euthyroid T2DM patients and (2) to analyze the potential effects of metformin treatment on this pathological process.

## 2. Materials and Methods

### 2.1. Study Population

A total of 369 patients diagnosed with T2DM were enrolled in this study from the Department of Endocrinology of the First Affiliated Hospital (Anhui Provincial Hospital) of the University of Science and Technology of China (USTC) between July 2017 and September 2018. It was agreed that the requirement for informed consent be waived because this study was designed to collect available data from participants' medical records retrospectively. Patients were stratified into two groups according to abdominal ultrasonography: NAFLD and non-NAFLD groups. Diffuse fatty liver can be defined by the presence of at least two out of three abnormal findings on abdominal ultrasonography: diffusely increased liver near field ultrasound echo (“bright liver”), liver echo greater than kidney; vascular blurring; and the gradual attenuation of far field ultrasound echo [10]. These evaluations were performed independently by two different experienced doctors of ultrasound medicine. To further evaluate the effects of metformin on thyroid function, clinical data of 145 T2DM patients diagnosed with NAFLD were stratified into two groups according to whether metformin was used or not. Patients with acute complications of diabetes, hyperthyroidism, or hypothyroidism and patients with severe hepatic disease (if the value of liver function index exceeds the upper normal reference value by 1.5 times), severe CKD (defined as eGFR ≤ 60 mL/min/1.73 m^2^), cancer, or other severe coexisting illnesses were excluded from the study.

### 2.2. Clinical and Laboratory Evaluation

Age, gender, body mass index (BMI, kg/m^2^), duration of diabetes, use of hypoglycemic drugs (taken continuously over at least 3 months, metformin defined as more than 1.5 g/day), and basal metabolic rate (BMR) (estimated using the equation (systolic pressure − diastolic pressure + heart rate − 111, taking the average of three consecutive readings)) were obtained from participants' records. All patients were tested for biochemical markers of liver function: alanine transaminase (ALT) and aspartate transaminase (AST); kidney function: creatinine (Cr), estimated glomerular filtration rate (eGFR), calcium (Ca^2+^), and phosphorus (P); glucose metabolism: fasting blood glucose (FBG), fasting insulin (Fins), fasting c peptide (FCP), hemoglobin A1c (HbA1c), and homeostatic model assessment-insulin resistance (HOMA-IR); lipid metabolism: triglycerides (TG), total cholesterol (TC), low-density lipoprotein cholesterol (LDL-c), and high-density lipoprotein cholesterol (HDL-c); bone metabolism: osteocalcin, type I procollagen peptide, *β*-CrossLaps, and 25-hydroxyvitamin D (25-OHD); and thyroid function: free triiodothyronine (FT3, normal range: 3.28-6.47 pmol/L), free thyroxine (FT4, normal range: 7.90-19.05 pmol/L), and TSH (normal range: 0.350-4.949 mIU/L), FT3/FT4 ratio at baseline. TSH, FT4, and FT3 were measured by electrochemiluminescent immunoassays on a Roche Modular E170 analyzer, using kits provided by the manufacturer (Roche, Mannheim, Germany).

### 2.3. Statistical Analyses

IBM SPSS Statistics ver. 22.0 (IBM Co., Armonk, NY, USA) was used. Continuous measurements, such as the mean (SD), were utilized if data were normally distributed; however, if the data were not normally distributed, the median (IQR) was used. Categorical variables were described by frequency and percentages (%). Independent tests, including the *t*-test, chi-square test, or Mann–Whitney *U* test, were used to compare the two patient groups. Logistic regression analysis was used to calculate odds ratios (ORs) and their 95% confidence intervals (CIs) for the risk of NAFLD while adjusting for potential confounding variables. Spearman's correlational analysis was used to describe the relationship between thyroid index, BMI, and liver function among T2DM subjects with NAFLD. Statistical significance was set at *p* < 0.05.

## 3. Results and Discussion

### 3.1. Demographic and Metabolic Characteristics of Study Subjects

The data of 369 T2DM patients (64.5% male and 39.3% NALFD) were evaluated. The mean age of the study sample was 55.26 ± 12.18 years, ranging from 33–72 years. The duration of type 2 diabetes ranged between 0 and 16 years. The patients in the NAFLD group had significantly lower age, duration of diabetes, and higher BMI, BMR, ALT, AST, P, FIns, FCP, TG, TC, LDL-c, FT3, FT3/FT4 ratio, and TSH compared with those in the non-NAFLD group (all *p* < 0.05). There were no significant differences in sex, Cr, eGFR, P, FPG, HbA1c, HOMA-IR, HDL-c, and FT4 between the two groups (all *p* > 0.05) (Table 1).

### 3.2. Adjusted Odds Ratios for NAFLD Risk

The multivariate logistic regression model was used to analyze the risk factors of NAFLD. After adjusting for all factors with significant associations emerging from the univariate analysis, the odds of NAFLD in T2DM patients increased with each unit increase in BMI, FT3, and TSH; ORs were 1.252, 3.020, and 1.581, respectively (all *p* < 0.05) (Table 2).

### 3.3. Association among Thyroid Index, BMR, BMI, HOMA-IR, and Liver Function

Table 3 shows positive correlations between ALT and FT3 levels (*r* = 0.221, *p* = 0.010) and BMI and HOMA-IR (*r* = 0.586, *p* < 0.001) and negative correlations between TSH and BMR (*r* = −0.618, *p* < 0.001) and FT3 and BMR (*r* = −0.452, *p* < 0.001) among T2DM subjects with NAFLD.

### 3.4. Metabolic Characteristics of Type 2 Diabetes Patients with NAFLD, by Metformin Use

Based on metformin use, 145 T2DM patients with NAFLD were stratified into two groups (not-used group and used group). Both groups were similar in terms of age and gender distribution. The not-used group had higher values of HOMA-IR, FT3, TSH, and BMI (both *p* < 0.05). The general characteristics, biochemical markers, and thyroid indices of the two groups are summarized in Table 4.

### 3.5. Thyroid Indices in Groups Defined by Metformin Use, Stratified by BMI

**Figure 1:**
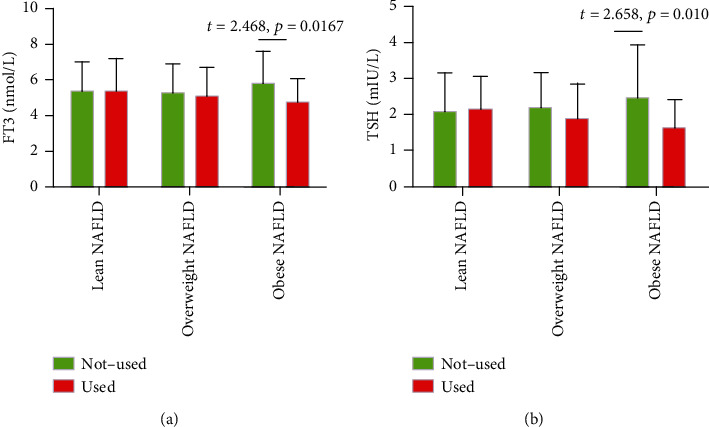
Comparison of serum concentrations of FT3 (a) and TSH (b) between NAFLD patients who used and did not use metformin, stratified by BMI.

The usage of metformin may be influenced by both thyroid metabolism and BMI. An additional analysis was performed to compare serum concentrations of FT3 and TSH among BMI-based groups of T2DM patients with NAFLD. Patients were stratified into three groups according to Chinese BMI characteristics: lean (BMI ≤ 24.0 kg/m^2^, *N* = 34), overweight (BMI > 24.0 ≤ 28.0 kg/m^2^, *N* = 62), and obese (BMI > 28.0 kg/m^2^, *N* = 49). In the nonuse of metformin patients, our outcomes showed statistical elevation (*p* < 0.05) of TSH and FT3 in the obese group compared to the two other groups. However, no statistical difference was found in the use of metformin patients. A significant difference in serum FT3 (*t* = 2.468, *p* = 0.0167) and TSH (*t* = 2.658, *p* = 0.010) levels was found between obese NAFLD patients that used and did not use metformin. However, this positive effect of metformin was not detected in lean and overweight NAFLD patients (both *p* > 0.05) (Figure 1).

## 4. Discussion

Nowadays, due to the rapidly increasing incidence of obesity and obesity-related diseases, NAFLD has become a significant public health problem because it is associated with important cardiovascular and metabolic risk factors [11]. NAFLD is generally considered to be the liver manifestation of the metabolic syndrome and is more common among people with type 2 diabetes [12]. In Japan, liver-related diseases, such as cirrhosis and hepatocellular carcinoma, are now the third leading cause of death in T2DM, which is closely associated with NAFLD [13]. The main pathological change caused by T2DM in the Chinese population is insulin resistance. This condition leads to increased migration of fatty acids to the hepatic cells, which accelerates the progression of NAFLD [5]. In a systematic review by Mantovani et al., NAFLD was present in 50%–75% of T2DM patients, with variation according to ethnicity [14]. A number of researchers have explored risk factors associated with relevant hypothyroidism (serum TSH concentration within the upper normal range) and NAFLD [15]. Both T2DM and impaired thyroid function can increase the risk of existing NAFLD progressing to NASH, as well as the subsequent development of cirrhosis [16]. However, few studies have explored the association between thyroid function and NAFLD in euthyroid T2DM patients. Since the T2DM patients have a high incidence of NAFLD as well as an increased risk of harmful clinical outcomes, the potential mechanism in this pathological process and its targeted treatment should be studied.

Thyroid hormones coordinate a diverse array of physiological events, including homeostasis and energy maintenance, and thyroid dysfunction is associated with transformations in body weight and its distribution and body composition [17]. A meta-analysis of 44,140 individuals indicated that hypothyroidism was associated with an increased risk of NAFLD independently of age, sex, BMI, and other known risk factors [14]. Liu et al. showed that TSH (OR = 1.108) and FT3 (OR = 1.258) levels were independently associated with the risk of NAFLD (diagnosed by ultrasound) [18]. Euthyroid subjects with suspected NAFLD had higher FT3, lower FT4, and higher FT3/FT4 ratios, which may be indicators of central obesity [8]. Kim et al. reported that biopsy-confirmed fibrosis in NAFLD is strongly associated with elevated TSH levels in a dose-dependent way, even within the normal thyroid reference range [7]. Our study also found statistically higher TSH and FT3 levels in T2DM patients with NAFLD than in patients without NAFLD. BMR and HOMA-IR are frequently used to estimate the energy metabolism statement/insulin resistance, as they can be easily calculated. Our research indicates that BMR was negatively correlated with TSH and FT3 levels; however, HOMA-IR had correlations in the opposite direction. These results suggest that T2DM complicated with NAFLD has a thyroid hormone resistance-like manifestation, i.e., relevant hypothyroidism, that may play a vital role in the pathological process of NAFLD.

As previously mentioned, the environment of insulin resistance and reduced TH signaling may be involved in the pathological process of NAFLD. Therefore, it is reasonable to explore medicines to treat these pathophysiological features. AMP-activated protein kinase (AMPK) is an energy sensor that controls cellular metabolism, and its activation results in enhanced insulin sensitivity [19]. Thyroid hormone accelerates energy metabolism in the liver by upregulation of AMPK, thus supporting a higher energy expenditure condition [20]. Given its vital role in both insulin resistance and thyroid metabolism, the AMPK pathway should be extensively studied as a potential new therapeutic target in T2DM complicated with NAFLD. It is an established fact that metformin, the most widely used antidiabetic drug, attenuates T2DM-induced insulin resistance by acting on the AMPK pathway [21]. Huang et al. found that metformin treatment decreased liver lipid accumulation by inhibiting the expression of ROCK1, leading to the activation of the downstream AMPK pathway [11]. A sizeable body of literature on metformin's effects on thyroid function comprises mixed results, with a probability of a modest decrease in TSH level, but no consistent signal for this decline. In a prospective study, increased metformin use was associated with low TSH levels among patients with treated hypothyroidism but not those with normal thyroid function [22]. A study by Al-Alusi et al. shows that metformin suppressed serum TSH levels independent of increasing levothyroxine absorption [23]. Cappelli et al. found a significant decrease in TSH levels in euthyroid patients with higher baseline TSH concentrations after metformin therapy [9]. In the nonuse of metformin patients, our outcomes showed an elevation of TSH and FT3 in the obese group compared to the two other groups. However, the mechanism involved in the increase in TSH and FT3 levels in obese patients is unclear. Gokosmanoglu et al. postulate that the underlying mechanism in TSH elevation is the development of resistance in TH receptors in target tissues of obese patients that reduces the effectiveness of the hormone in target tissues [24]. The study of Laclaustra has demonstrated that obese individuals with a steady consumption of high-calorie foods have an elevated risk of thyroid hormone resistance [25]. Resistance to thyroid hormone may reflect energy balance problems driving type 2 diabetes and NAFLD [26]. Our study results also show that obese NAFLD patients that used metformin had remarkably lower serum FT3 and TSH levels than those who did not use metformin. However, this advantage of metformin was not observed among lean and overweight NAFLD patients, indicating that obese patients had more severe energy balance problems. This study had several limitations. First, as this was a single-center study in China, the results might not be directly applicable to other ethnicities and regions. Second, ultrasonography has limited sensitivity and does not reliably detect steatosis when <20% or in individuals with high BMI (>40 kg/m^2^), which might have led to potential heterogeneity. Finally, the validity of the findings cannot be ascertained due to unmeasured confounding factors.

## 5. Conclusions

The pathological mechanism of T2DM complicated with NAFLD in euthyroid patients may be associated with not only insulin resistance but also a thyroid hormone resistance-like manifestation, i.e., relevant hypothyroidism. Metformin can potentially decrease the double-resistance situation, especially in obese individuals. Given the importance of this result, further studies are warranted to understand better the pathological process and targeted treatment options in these patients.

## Figures and Tables

**Table 1:** Demographic and metabolic characteristics of study subjects by NAFLD status.

Characteristics	Non-NAFLD (*N* = 224)	NAFLD (*N* = 145)	*T*/*χ*2/*F* value	*p* value
General				
Male (*N*, %)	143 (64.13%)	95 (65.52%)	0.108	0.742
Age (years)	56.77 ± 12.01	53.06 ± 12.67	2.832	0.005
BMI (kg/m^2^)	23.72 ± 3.37	26.92 ± 4.04	-7.233	<0.001
BMR (%)	15.12 ± 6.45	11.68 ± 6.37	6.654	<0.001
Duration of diabetes (years)	8 (3, 14)	6 (1.75, 10)	-2.043	0.041
Biochemical markers				
ALT (IU/L)	19 (14, 28)	24 (15.5, 40)	-3.380	0.001
AST (IU/L)	19 (16, 25)	21 (16, 30.5)	-1.949	0.051
Cr (*μ*mol/L)	76.11 ± 17.94	77.95 ± 14.65	-1.013	0.312
eGFR (mL/min/1.73 m^2^)	104.21 ± 22.31	101.04 ± 18.93	0.421	0.632
Ca^2+^ (mmol/L)	2.27 ± 0.16	2.28 ± 0.15	-1.044	0.297
P (mmol/L)	1.15 ± 0.20	1.21 ± 0.18	-2.907	0.004
Glucose metabolism				
FBG (mmol/L)	8.75 ± 3.95	9.10 ± 4.05	-0.621	0.535
FCP (nmol/L)	0.35 ± 0.19	0.49 ± 0.25	-3.968	<0.001
HbA1c (%)	8.72 ± 2.29	8.82 ± 2.18	-0.399	0.690
Fins (pmol/L)	48.85 (33.62, 81.64)	66.05 (46.12, 112.62)	-2.983	0.003
HOMA-IR	2.75 (1.34, 5.21)	3.64 (1.59, 6.52)	-1.892	0.059
Use of hypoglycemic drugs				
Insulin (*N*, %)	68 (30.36%)	39 (26.90%)	0.512	0.474
Metformin (*N*, %)	97 (43.30%)	79 (54.48%)	4.410	0.036
Thiazolidinediones (*N*, %)	23 (10.27%)	18 (12.41%)	0.410	0.522
Sulfonylureas/glinides (*N*, %)	102 (45.54%)	65 (44.83%)	0.279	0.597
DPP − 4i (*N*, %)	47 (20.98%)	27 (18.62%)	0.306	0.580
Glucosidase inhibitors (*N*, %)	101 (45.09%)	71 (48.97%)	0.531	0.446
Lipid metabolism				
TG (mmol/L)	1.40 (1.03, 1.97)	2.09 (1.59, 3.07)	-7.195	<0.001
TC (mmol/L)	4.44 ± 1.09	4.93 ± 1.23	-3.814	<0.001
LDL-c (mmol/L)	2.33 ± 0.78	2.61 ± 0.79	-3.212	0.001
HDL-c (mmol/L)	1.05 ± 0.29	1.08 ± 0.90	-0.384	0.701
Bone metabolism				
Osteocalcin (ng/mL)	15.65 ± 5.95	14.62 ± 5.53	1.422	0.156
Type I procollagen peptide (ng/mL)	43.91 ± 18.28	39.99 ± 15.05	1.068	0.289
25-OHD (ng/mL)	18.26 ± 6.75	18.11 ± 6.74	0.194	0.846
*β*-CrossLaps (pg/mL)	413.7 (310.9, 664.00)	393.5 (249.8, 562.7)	-1.462	0.144
Thyroid metabolism				
FT3 (pmol/L)	4.96 ± 0.92	5.36 ± 1.60	-2.969	0.003
FT4 (pmol/L)	13.21 ± 3.86	13.07 ± 4.63	0.297	0.767
FT3/FT4 ratio	0.39 ± 0.08	0.43 ± 0.26	-2.401	0.017
TSH (mIU/L)	1.89 ± 1.11	2.09 ± 1.02	-1.778	0.046

Data are *N* (%), mean ± SD, or median (interquartile range). BMI: body mass index; BMR: basal metabolic rate; ALT: alanine aminotransferase; AST: aspartate transaminase; Cr: creatinine; eGFR: estimated glomerular filtration rate; Ca2+: calcium; P: phosphorus; FBG: fasting blood glucose; Fins: fasting insulin; FCP: fasting c peptide; HbA1c: hemoglobin A1c; HOMA-IR: homeostatic model assessment-insulin resistance; DPP-4i: dipeptidyl peptidase 4 inhibitors; TG: triglycerides; TC: total cholesterol; LDL-c: low-density lipoprotein cholesterol; HDL-c: high-density lipoprotein cholesterol; FT3: free triiodothyronine; TSH: thyroid stimulating hormone; FT4: free thyroxine; NAFLD: nonalcoholic fatty liver disease.

**Table 2:** Adjusted odds ratios for NAFLD risk.

	Estimate (*B*)	Standard error	Wald statistic	OR (95% CI)	*p* value
BMI	0.255	0.065	12.020	1.252 (1.103, 1.422)	0.001
FT3	1.105	0.422	6.855	3.020 (1.320, 6.908)	0.009
TSH	0.458	0.217	4.460	1.581 (1.034, 2.419)	0.035

BMI: body mass index; FT3: free triiodothyronine; TSH: thyroid stimulating hormone; OR: odds ratio; CI: confidence interval.

**Table 3:** Correlations between thyroid index, BMI, and liver function among T2DM subjects with NAFLD.

	BMR	ALT	HOMA-IR
BMI	0.164 (0.069)	0.158 (0.083)	0.586 (<0.001)
FT3	-0.452 (<0.001)	0.221 (0.010)	0.093 (0.378)
TSH	-0.618 (<0.001)	-0.108 (0.213)	0.124 (0.159)

Correlation coefficients between variables (*r*) and their corresponding *p* values are expressed as “*r* (*p* value).” BMI: body mass index; FT3: free triiodothyronine; TSH: thyroid stimulating hormone; ALT: alanine aminotransferase; HOMA-IR: homeostatic model assessment-insulin resistance.

**Table 4:** Metabolism characterization of type 2 diabetes with NAFLD, grouped by metformin usage.

Characteristics	Not-used metformin (*N* = 66)	Used metformin (*N* = 79)	*T*/*χ*2/*F* value	*p* value
General				
Male (*N*, %)	44 (66.67%)	51 (64.56%)	0.071	0.790
Age (years)	52.14 ± 13.01	53.79 ± 12.28	0.784	0.434
BMI (kg/m^2^)	27.14 ± 4.22	24.65 ± 3.29	3.903	<0.001
BMR (%)	12.05 ± 6.78	11.54 ± 6.21	0.472	0.637
Duration of diabetes (y)	5 (2, 9)	6 (2, 10.5)	0.872	0.513
Use of hypoglycemic drugs				
Insulin (*N*, %)	17 (25.76%)	21 (26.58%)	0.013	0.910
Thiazolidinediones (*N*, %)	10 (15.15%)	8 (10.13%)	0.835	0.361
Sulfonylureas/glinides (*N*, %)	34 (51.52%)	31 (39.24%)	2.191	0.139
DPP − 4 inhibitors (*N*, %)	15 (22.73%)	12 (15.19%)	1.348	0.246
Glucosidase inhibitors (*N*, %)	36 (54.55%)	35 (44.30%)	1.509	0.219
Biochemical data				
ALT (IU/L)	22 (14, 35)	25.5 (16, 41)	-1.681	0.134
HOMA-IR	3.59 (1.62, 6.61)	3.20 (1.36, 5.48)	2.092	0.011
eGFR (mL/min/1.73 m2)	106.23 ± 19.35	100.29 ± 18.47	1.887	0.061
HbA1c (%)	8.93 ± 2.64	8.71 ± 2.13	0.555	0.580
Thyroid metabolism				
FT3 (pmol/L)	5.68 ± 1.70	5.14 ± 1.45	2.064	0.041
FT4 (pmol/L)	14.19 ± 3.90	13.02 ± 4.69	1.613	0.109
FT3/FT4 ratio	0.44 ± 0.28	0.41 ± 0.15	0.8219	0.413
TSH (mIU/L)	2.25 ± 1.13	1.91 ± 0.94	1.978	0.049

Data are *N* (%), mean ± SD, or median (interquartile range). BMI: body mass index; BMR: basal metabolic rate; ALT: alanine aminotransferase; eGFR: estimated glomerular filtration rate; HbA1c: hemoglobin A1c; HOMA-IR: homeostatic model assessment-insulin resistance; DPP-4i: dipeptidyl peptidase 4 inhibitors; FT3: free triiodothyronine; TSH: thyroid stimulating hormone; FT4: free thyroxine; NAFLD: nonalcoholic fatty liver disease.

## Data Availability

The datasets analyzed during the current study are available from the corresponding author.
